# Crisis and Emergency Risk Communication: Lessons from the Elk River Spill

**DOI:** 10.1289/ehp.122-A214

**Published:** 2014-08-01

**Authors:** John Manuel

**Affiliations:** John Manuel of Durham, NC, is a regular contributor to EHP and the author of *The Natural Traveler Along North Carolina’s Coast* and *The Canoeist*.

**Figure:**
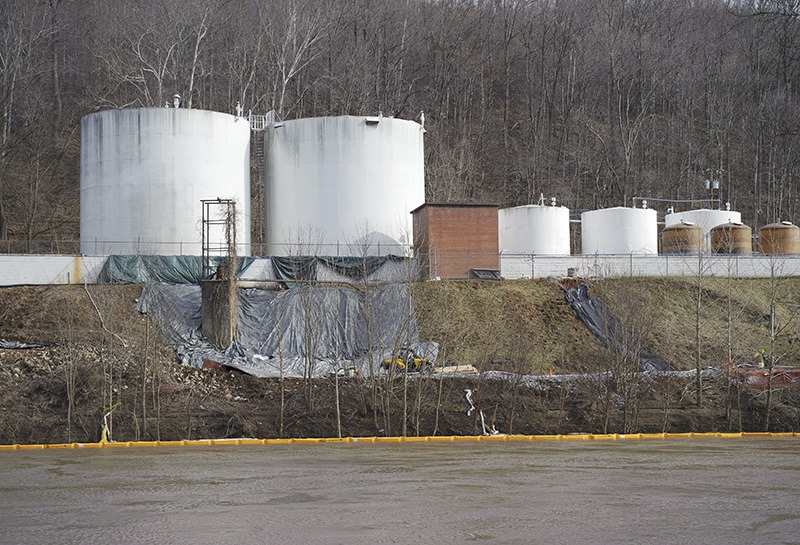
Inspectors first discovered chemical mixtures leaking from a Freedom Industries storage tank into the Elk River on 9 January 2014. © AP Photo/Steve Helber, File

Freedom Industries’ Charleston, West Virginia, tank farm sits on a narrow crescent of flat land between a steep, wooded hillside and the green waters of the Elk River. On 9 January 2014 inspectors discovered a stream of chemical mixture leaking from the bottom of tank 396, under a containment wall, and into the river, which provides drinking water for some 300,000 residents.^1^^,^^2^ The mayhem that followed the discovery of the leak rocked the Mountain State. Within two weeks nearly 600 people visited emergency departments for symptoms claimed to be related to the spill, and 13 were hospitalized.^3^ Schools and businesses closed. According to a preliminary study by the Marshall University Center for Business Research, the spill cost Charleston-area businesses more than $61 million in the first month alone.^4^

But the event has also carried a heavy intangible cost. By all accounts, the spill caught everyone off guard. Public health officials, hamstrung by a lack of toxicity data, scrambled to assess the potential for harm among exposed residents. Efforts to communicate their progress to the general public were not always successful; terms that mean one thing in a risk-assessment context meant something completely different to tense residents awaiting direction. The public faith was sorely tested by what Charlestonians and other observers perceived as inaccurate, conflicting, and in some cases nonexistent communications from officials.

As a result, the Elk River spill has won the reputation of “a case study in what not to do in terms of risk communication,” says Rahul Gupta, executive director of the Kanawha–Charleston Health Department. “There is a lot that everyone can learn from this event.”

## Crisis and Emergency Risk Communication

We humans have no doubt been warning each other of hazards for as long as our kind have existed. However, the institutional practice of risk communication by government and industry is a relatively recent phenomenon. David Ropeik, an instructor in the Harvard University Extension School’s Environmental Management Program, traces this practice to the late 1970s and the rise of public protests over the chemical and nuclear industries.

**Figure:**
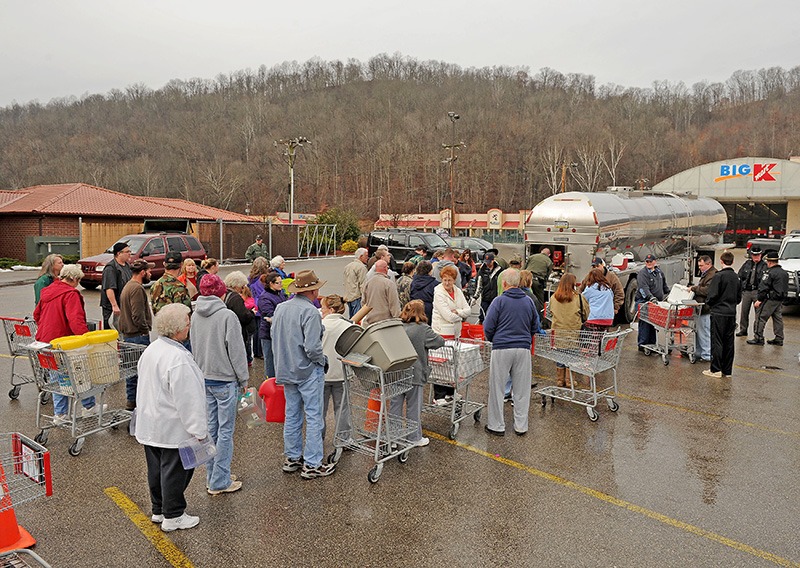
The Elk River provides drinking water for some 300,000 Charleston-area residents, who for several days relied on water that was trucked in by the National Guard and other groups. © AP Photo/Tyler Evert

**Figure:**
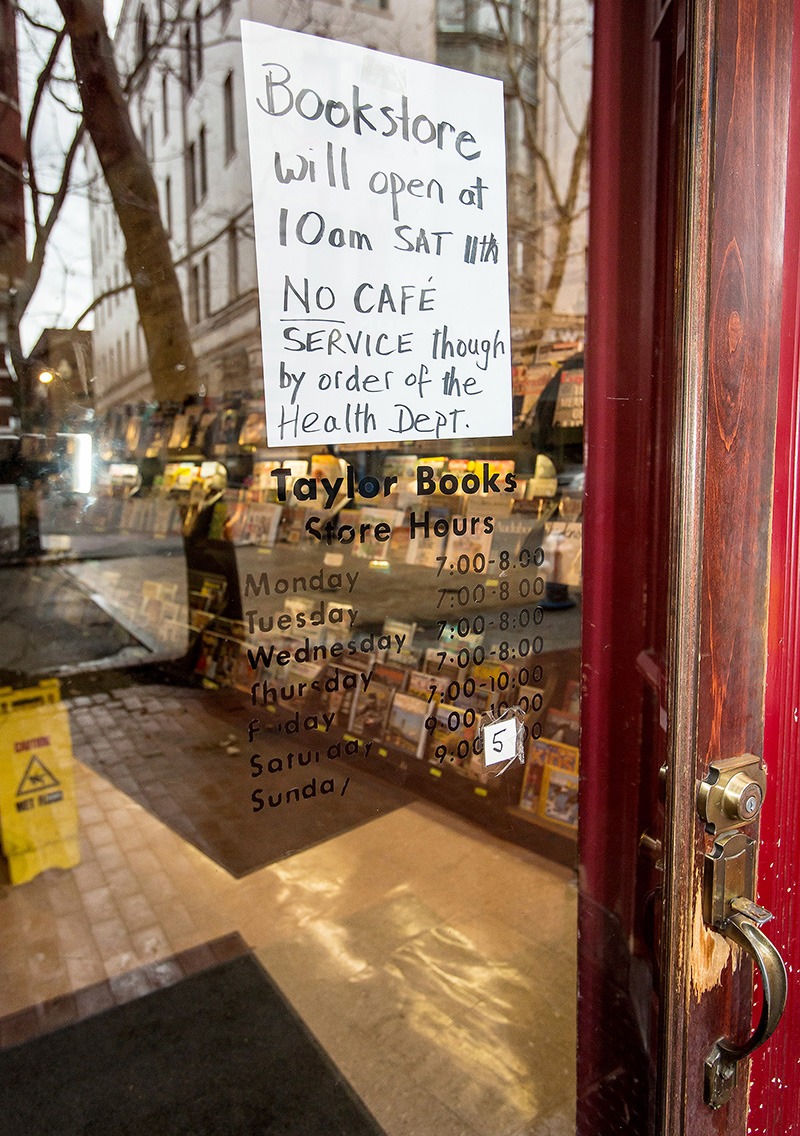
The disruption cost local businesses more than $61 million, according to preliminary estimates. © AP Photo/Michael Switzer

“These companies and their regulators went to people like [researchers] Paul Slovic and Peter Sandman to ask why the public was reacting ‘irrationally’ to their products and practices,” Ropeik says. “They wanted to know what they could do to make these people calm down.”

But the researchers didn’t offer ways to quiet the public. They didn’t suggest that people could be persuaded to relinquish their fears just by being plied with more facts. Instead, they talked about the role of people’s emotions in their response to risk. They explained that everyone—rich, poor, educated, and otherwise—has an emotional response when confronted with risk, and that this reality must be acknowledged and dealt with in any communications about a potential threat.^5^^,^^6^ Today, risk communication is a widely recognized field with numerous theories, tools, and best practices.

Properly speaking, the communications issued following the Elk River spill fall under the larger rubric of crisis and emergency risk communication (CERC). Crisis communication is what happens as an event is unfolding and in the immediate aftermath—one example is the do-not-use order issued by West Virginia American Water following the discovery of the contamination. Discussion about potential long-term health impacts from chemical exposures, on the other hand, would be considered risk communication. Ropeik says many environmental crises, particularly those involving chemical spills, include elements of both crisis and risk communication.

Recognizing the importance of training public health officials in these skills, the Centers for Disease Control and Prevention (CDC) in 2002 published a thick manual addressesing a number of topics critical to successful public, partner, and stakeholder communication during crises and emergencies. Updated in 2012, the manual advises responders, “Today’s public and your stakeholders demand immediate and credible communication in real time during a crisis response”; by applying CERC principles, “you can learn what to say, when to say it, and how to say it to help you preserve or win the public’s trust. Most importantly, it can save lives.”^7^

## Six Principles

The CERC manual begins by defining six principles of effective crisis and risk communication. These are 1) be first, 2) be right, 3) be credible, 4) express empathy, 5) promote action, and 6) show respect. Crises are time sensitive, and quick communication of relevant information is crucial to save lives and address public fears; hence, the directive to “be first.” In the Elk River spill, the first to report something amiss were local citizens. But it was hours before officials publicly addressed the situation, warning people not to drink the water.

**Figure:**
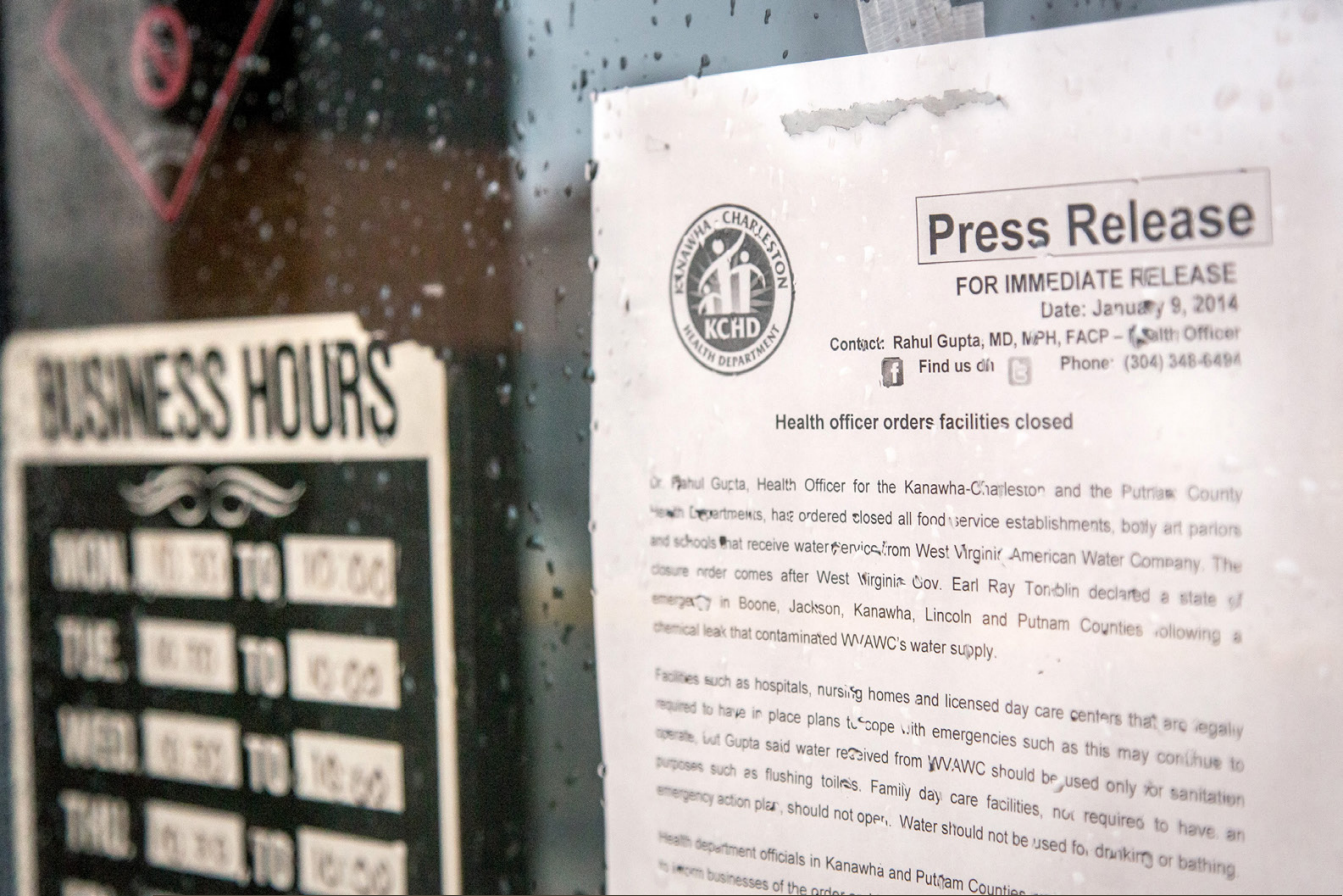
Many residents found Rahul Gupta, of the local health department, the most trustworthy of all the officials involved in the spill response. Gupta says the public “viewed us as trying to genuinely help people instead of referring them to another entity. We continued engagement on a daily basis with timely information in an unbiased, transparent manner.” © AP Photo/Michael Switzer

Calls complaining of a strong licorice smell near the Freedom Industries tank farm started coming in to the West Virginia Department of Environmental Protection (WVDEP) around 8:15 the morning of January 9. WVDEP sent two inspectors to the site. They consulted with a Freedom Industries employee who initially told them there was no problem. But the inspectors quickly discovered a chemical stream leaking from one of the tanks. The employee resisted calling in the spill to WVDEP (as required by law) until ordered to do so—just before noon—by department inspectors. The leaking material, he said, was crude MCHM, a chemical mixture used to separate rock and clay from coal.^2^

In testimony presented to the state Public Service Commission, West Virginia American Water official Jeffrey McIntyre reported that the utility was notified of the spill around the same time and began sampling raw and treated water soon thereafter; crude MCHM was first detected in treated water at the plant a little after 4:00 p.m. The utility issued the do-not-use order at 5:50 p.m., and officials first addressed the public at a 6:00 p.m. news conference and later via social media and automated telephone calls to customers.^8^

By then, people had been drinking the water all day. On local news anchor Elizabeth Noreika’s Facebook page, anxious viewers—unaware of the steps the utility was taking to test the water—posted comments such as “The leak happened when & they’re just now deciding this? Everyone in my house has already bathed in it today, including my 3 month old daughter. I’ve washed her bottles in it. My animals drank it. Ughhh. My babies better not get sick!”^9^

The second principle of effective crisis communication is “be right.” Freedom Industries failed in that regard; when asked by the WVDEP operator on the morning of the spill if the chemical had reached the Elk River, the employee said no. He also said the MCHM mixture was not hazardous.^2^ In one sense that’s true; WVDEP spokeswoman Kelley Gillenwater confirms that crude MCHM is not regulated as a transportation hazard under federal or international shipping regulations. However, the mixture’s Material Safety Data Sheet (MSDS) warns that the compound can be harmful if swallowed and may cause irritation of the eye, skin, and respiratory tract.^10^

Freedom Industries also fell short of the third principle, “be credible.” A company spokesperson first estimated that 1,000–2,500 gallons of MCHM had leaked from the tank.^9^ Days later that estimate was increased to 7,500 and then 10,000 gallons.^11^ Ropeik comments, “If the first estimate was clearly stated as an estimate, the communicator left the wiggle room for later information, which is standard crisis and risk communication good practice.” But it’s not clear that this nuance was ever conveyed to the public. Each member of the Charleston general public interviewed for this article cited the continually rising estimates as a reason for mistrust.

Furthermore, the company originally said only one chemical mixture, crude MCHM, had been involved in the leak, although employees reportedly knew the tank contained a second chemical mixture, PPH, comprising two propylene glycol phenyl ethers; that information was not communicated until nearly two weeks later.^12^

“Having this revelation so late in the game is completely unacceptable,” said Randy Huffman, WVDEP cabinet secretary, in a January 22 press release. “We have ordered Freedom to reveal any other information they have regarding the contents of the tank that leaked. Having to order them to provide such obvious information is indicative of the continued decline of their credibility.”^13^

The fourth principle of effective communication is “be empathetic.” In his one televised public appearance, Freedom Industries president Gary Southern apologized for the spill, then attempted to end the interview, saying, “It’s been an extremely long day.”^14^ Although Southern did go on to answer further questions for journalists, angry viewers felt his behavior was insensitive.^15^ The company’s case was not helped by the girlfriend of its CEO, who reportedly wrote on her public Facebook account (since closed) that “no one and no thing, has been harmed due to this leakage.”^16^ Freedom Industries’ public relations disasters piled up to the point that on January 12 the company’s own PR firm, Charles Ryan Associates, quit.^17^ Shortly thereafter, Freedom Industries declared bankruptcy^18^ and has not talked to the media since.

## Federal Communications

State and federal agencies also came in for their share of criticism with regard to their communications in the wake of the spill. The greatest controversy had to do with communications about a “safe level” of MCHM in drinking water.

**Figure:**
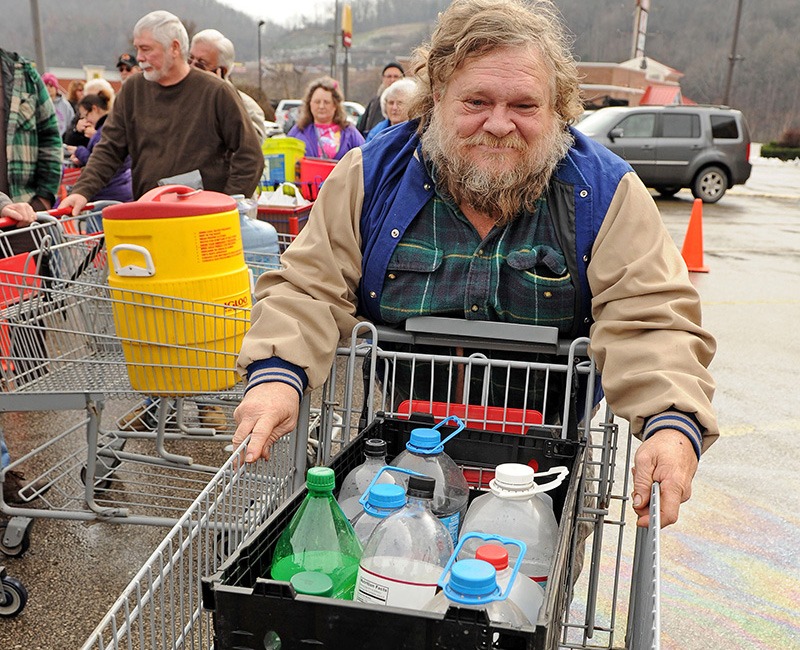
Effective communication before crises happen can play a vital role in educating the public about the presence of hazards and preparing them for the possibility that accidents may occur. It can also help industries, responding agencies, and the public better manage crises that do occur. © AP Photo/Tyler Evert

After the do-not-use order was issued the evening of January 9, the West Virginia Department of Health and Human Resources (DHHR) contacted the CDC seeking its help in determining the human health hazard presented by the contaminated drinking water.^19^ By law, states must take the initiative to request federal assistance in a disaster situation if it becomes apparent the emergency outstrips state resources; federal agencies do not have the authority to step in uninvited.^20^

The federal agency immediately began searching for information on the toxicity of crude MCHM. As with many of the 80,000-odd chemicals in use today, there are minimal data on crude MCHM’s effects on human health. The only information the CDC could find was contained in the MSDS provided by Eastman Chemical Company.^10^ According to Christopher Weis, toxicology liaison for the National Institute of Environmental Health Sciences, by the evening after the spill, officials at the National Library of Medicine had advised the CDC that, aside from the MSDS, there was no publicly available information about MCHM in their considerable holdings on chemical safety.^21^

Despite the dearth of data, CDC emergency response staff were charged with estimating a measure by which drinking water could be screened for safety. They started with the one piece of data they had: the LD_50_ listed on the MSDS.^22^ The emergency responders then used standard risk assessment assumptions to estimate how that figure would translate to humans and to account for uncertainty. They arrived at 1 ppm as a threshold level for humans to safely consume.^23^

That number was relayed back to West Virginia officials, and state and federal officials repeatedly cited that number as a level believed to be without acute effects. Starting January 13, West Virginia American Water began screening water samples throughout its distribution system and lifting its do-not-use order for those areas that tested below 1 ppm crude MCHM.

What wasn’t clearly communicated to the public were the limitations of the screening level. For instance, early in the crisis Richard Denison, lead senior scientist with the Environmental Defense Fund, pointed out in a blog post that the manufacturer’s MSDS lacked any information about chronic health impacts, and that the screening level did not specifically account for the lack of data on exposure by inhalation or dermal contact. He further questioned the way in which officials applied uncertainty factors. Denison concluded, “Now, let me be clear. I am *not* saying that the level of 1 ppm is unsafe. I am saying that we have no way of knowing whether or not it is safe. The data needed to make that assessment simply do not exist for this chemical.”^24^

Eventually, however, more information did come to light. Federal agencies’ ongoing search for data on crude MCHM led them to proprietary studies held by the manufacturer.^25^ Once those studies were obtained, an expert workgroup comprising scientists from the National Institute of Environmental Health Sciences/National Toxicology Program, the National Library of Medicine, the Environmental Protection Agency, and the CDC/Agency for Toxic Substances and Disease Registry reviewed the studies and the methodology, and concurred that the 1-ppm short-term screening level was an appropriate safeguard.^23^

## Public Deals with Confusing Messages

Within a few days of lifting the do-not-use order, 150,000 customers had been cleared by West Virginia American Water to drink the water, according to utility spokeswoman Laura Jordan. But on January 15 the West Virginia Bureau for Public Health announced that pregnant women should continue to drink bottled water until crude MCHM was no longer detectable in tap water, “out of an abundance of caution,”^26^ a warning that left the public confused and worried.

When hundreds of people began showing up at area hospitals with symptoms they worried might be connected to the contaminated water, the Governor’s office hosted a press call at which State Health Officer Letitia Tierney reminded citizens they were in the middle of flu season and suggested their symptoms could be due to flu or anxiety.^27^ This statement infuriated Anne Berry, a Charleston family physician with two young children.

“From the beginning, we’ve been disappointed and angry because a lot of the public messages put out by the Governor, DHHR, DEP, and West Virginia American Water have been insulting,” Berry says. “They said, ‘Don’t worry. The water is safe.’ But the information doesn’t exist to say that definitively. We could smell the odor in our water for weeks.”

The one official Berry and others say they do trust is Gupta, of the Kanawha–Charleston Health Department. Gupta and other staff from the health department showed up at public meetings, where they listened to people’s concerns and tried to answer their questions. The meetings typically started out with an angry crowd, as townspeople sought answers. “It was important not to become offended and to remain professional,” Gupta says. “Once we explained everything we knew and shared their concerns, and expressed that we, too, would like to have more answers in the long term, people mostly felt better.”

Gupta says the health department took plenty of heat during these meetings, but he felt it was important for the public to understand what his agency did and didn’t know. “The best communication strategy was listening to people, sharing their concerns in an honest and forthright manner, and not being defensive or appearing to have ulterior motives,” he says. “They also viewed us as trying to genuinely help people instead of referring them to another entity. We continued engagement on a daily basis with timely information in an unbiased, transparent manner.”

The most significant lesson learned, Gupta says, was to be upfront with the public about the unknowns in a disaster. “We found that, generally, people want to trust their government in times of crisis,” he says. “It is the government who often provides them reason not to.” At one meeting he publicly refuted the suggestion that people’s symptoms were nothing more than the flu, saying, “We obviously cannot explain away [symptoms] by saying it’s the flu or something else. At this time, we shouldn’t even try to.”^28^

The potential for confusing public messages can be headed off to some extent even before crises occur. In addition to listing principles for effective crisis and risk communication, the CERC manual defines five stages of the communication lifecycle—pre-crisis, initial, maintenance, resolution, and evaluation. Effective communication during the initial pre-crisis stage can go a long way toward preparing the public for the possibility of disaster and setting the tone for communications during an actual emergency.^7^

During this first stage, the communicator’s job is to monitor and recognize emerging risks, educate the general public about those risks, and prepare the public for the possibility of an adverse event. With respect to the risk posed by the Freedom Industries tank farm along the Elk River, local, state and federal regulators dropped the ball on all counts. Despite requirements under the federal Emergency Planning and Community Right-to-Know Act,^29^ the local emergency planning committee had made no efforts to publicly identify facilities holding more than 10,000 pounds of hazardous chemicals.

These same emergency planners had no strategy for dealing with a chemical spill, despite the tank farm’s location just 1.5 miles above West Virginia American Water’s drinking water intake. Six years earlier, the U.S. Chemical Safety Board had recommended implementation of such a strategy following a chemical explosion at a nearby Bayer CropScience facility.^30^ If implemented, Gupta says, perhaps such a strategy could have prevented much of the confusion following the Elk River spill.

## Preparing for Next Time

At the time of the spill, West Virginia law did not require any monitoring of the Freedom Industries facility or other aboveground chemical storage facilities. “As the state regulatory agency, WVDEP can only do what the law allows,” says Gillenwater, “and the law did not allow for regulation of these tanks.”

Since then, the state passed Senate Bill 373, also known as the “spill bill.”^31^ The new law calls for annual inspection of aboveground chemical storage tanks (underground tanks are covered under a previous law). It requires water utilities to create source-water protection plans that include information about nearby hazards that could contaminate water supplies. Utilities also must develop emergency response plans. Lastly, the bill includes long-term medical monitoring of residents impacted by the spill.

The CDC has also made strides to connect with Charleston-area residents. In April the agency partnered with the state DHHR to survey more than 200 households on the perceived impact of the spill, the best routes for channeling information to the public, and access to alternative sources of drinking water. A total of 171 households completed the survey. Over half the respondents thought TV was the most reliable source of information, while a few others cited the reliability of the Internet, social media, and word of mouth. However, 8% of respondents felt there was no reliable source of information whatsoever.^32^

Accidents like the one at Elk River have a way of making everyone look bad. Obscure chemical mixtures for which there was little human health information leaked into a major public water supply. Overnight, an agency was forced to make a judgment on potential health risks that would properly take years of carefully designed study to determine.

The CDC acknowledges that less-than-clear communication about what was known and not known about the possible health effects of the Elk River spill may have affected communities’ trust in government. In a statement provided to *EHP*, CDC representatives wrote, “We are committed to following the principles of effective crisis and emergency risk communication, including acknowledging what we know and don’t know, as we continue to work with other federal partners and West Virginia to better understand the health effects of the chemicals involved in this spill.”

In the latest step in that ongoing state–federal collaboration, CDC leaders Tom Frieden and Robin Ikeda met with West Virginia Senator Joseph Manchin and public health officials on July 23. During the meeting, the CDC agreed to provide assistance to West Virginia on surveillance and data collection to determine the most appropriate next steps. The National Toxicology Program also has pledged to conduct toxicological studies on MCHM and PPH. Results from those studies should be available within a year.^33^

West Virginia may now be better prepared for a similar event, but all across the country chemicals with unknown long-term health effects sit in tanks and railcars along waterways and populated areas. Ideally people living in the vicinity of these facilities would be well informed and would take an active role in understanding the risks and benefits of chemical industry activities, yet how would they find out this information, if it were even publicly available? Astute crisis and risk communication can play a vital role both in educating the public about these inherent risks and preparing them for the possibility of an accident. It can also help industries, agencies, and the public manage a crisis should it occur.

It’s ironic, perhaps, that Gupta, the bearer of bad news, came to be seen as the most trusted source by so many residents. Asked for his thoughts on what he has learned from the Elk River spill, Gupta thumbs through the CERC manual, stopping at the six principles of effective communication. “There’s one missing here—‘be honest’,” he says. “If you don’t know the answer, say so.”
